# Effect of prehabilitation before total knee replacement on postoperative patient-reported joint awareness, enablement and knee function: protocol for the PROTEKT randomised controlled trial

**DOI:** 10.1136/bmjopen-2025-113185

**Published:** 2026-03-03

**Authors:** Marcus Ljung, Kristin Gustafsson, Joanna Kvist

**Affiliations:** 1 Department of Health, Medicine and Caring Sciences, Unit of Physiotherapy, Linköping University, Linköping, Sweden; 2 Department of Orthopedics, Rehabilitation Unit, Vrinnevi County Hospital, Norrköping, Sweden; 3 Rehabilitation Centre, Ryhov County Hospital, Jönköping, Sweden

**Keywords:** Orthopedics, Physical Therapy Modalities, Exercise, Health Education

## Abstract

**Introduction:**

Knee osteoarthritis (OA) causes pain, reduced function and disability and may require total knee replacement (TKR). Although TKR is effective, up to 20% of patients remain dissatisfied, partly due to poor preoperative function and unrealistic expectations. Long waiting times for surgery may worsen patients’ function, yet preoperative physiotherapy is rarely offered. Prehabilitation—exercise and education before surgery—could improve postoperative recovery, but current evidence is limited. This trial investigates whether adding prehabilitation to standard care before TKR improves postoperative patient-reported joint awareness, enablement and knee function.

**Methods and analysis:**

This multicentre, randomised controlled parallel-group trial is planned to be conducted within two specialised orthopaedic outpatient rehabilitation units in the southeast healthcare region of Sweden. Eligible patients (40–85 years, awaiting unilateral TKR) are randomised 1:1, stratified by age (≤67, >67 years), to either 8 weeks of prehabilitation—comprising two times per week supervised exercise therapy (strength, range of motion and balance) and education—in addition to standard care, or to standard care alone. Standard care consists of self-care, a single standardised preoperative education session and standardised postoperative rehabilitation. Assessments are conducted at baseline, post-intervention, 1 week pre-surgery and 6, 12 and 52 weeks post-surgery. A total of 110 patients will be recruited to the trial. Primary outcomes are joint awareness (Forgotten Joint Score-12) and patient enablement (modified Patient Enablement Instrument-2). Secondary outcomes are patient satisfaction (5-category Likert scale), the Knee injury and Osteoarthritis Outcome Score, the EuroQol 5 Dimension 3 Level questionnaire, the International Physical Activity Questionnaire—short form, objective function and accelerometer-based physical activity. Analyses will follow intention-to-treat and per-protocol principles. Between-group and within-group differences will be tested using t-tests or non-parametric equivalents, and linear mixed models or generalised linear models. Multiple linear regression and logistic regression will be used to analyse predictor variables for the primary outcomes. Sensitivity analyses will be performed to quantify the magnitude of missing data from patients lost to follow-up.

**Ethics and dissemination:**

The trial has received ethical approval from the Swedish Ethical Review Authority (reg. no.2023-05120-01) and complies with the Declaration of Helsinki. Signed informed consent is collected for all patients before entering the trial. Results will be submitted for publication in a peer-reviewed journal and presented at international/national conferences. The findings may improve future clinical guidelines and care pathways for patients undergoing TKR.

**Trial registration number:**

NCT06290336.

**STRENGTHS AND LIMITATIONS OF THIS STUDY**

- The concealed, stratified randomisation reduces the risk of allocation bias.
- Supervision of exercise therapy ensures adequate intervention delivery and allows for easy monitoring of exercise technique, dosage and progression.
- Blinded assessors of objective outcomes reduce the risk of detection bias.
- The multicentre design promotes generalisation of trial results but increases risk of lower internal validity.
- Variation in waiting times for surgery between patients presents challenges for the statistical analysis.

## Introduction

 Osteoarthritis (OA) is a condition that affects the entire joint, with the knee being particularly vulnerable.^1^ The disease typically progresses over time, but symptoms such as pain, swelling, stiffness and reduced range of motion (ROM) can fluctuate. OA can result in disability, challenges with daily activities (eg, walking) and significant patient suffering.^1^ First-line treatment is non-surgical and includes patient education, activity modification, exercise therapy and pain management strategies.^2^,^4^ When these approaches are no longer sufficient, patients are often referred for assessment for joint replacement surgery.^5 6^ Total knee replacement (TKR) is the most common surgical intervention for knee OA, with approximately 19 000 procedures performed annually in Sweden.^7^ While TKR is generally a successful treatment, around 15–20% of patients report dissatisfaction 1–3 years after surgery.^7^,^9^ Reasons for dissatisfaction are multifactorial, including surgical complications, poor preoperative knee function and unrealistic expectations regarding rehabilitation and outcomes.^810^,^14^ Dissatisfaction with the surgery result is likely to indicate higher joint awareness in everyday life situations.^15 16^

In Sweden, the total waiting time, from primary care referral to orthopaedic consultation and subsequent surgery, can extend up to 12–24 months.^17^ Participation in exercise and physical activity tends to decline after first-line treatment,^18^ and this tendency may accelerate while awaiting surgery. Currently, routine physiotherapy management is rarely offered during this period, and prolonged waiting times may further reduce participation in exercise and physical activity, with subsequent loss of leg muscle strength and volume, and reduced ROM. This contributes to the deterioration of knee function, and these are factors associated with poorer outcomes after TKR.^810^,^12 19 20^ This supports the rationale for the hypothesis that improving knee function, physical activity and patient knowledge about OA and TKR before surgery, referred to as prehabilitation, may enhance postoperative results and bridge the gap between the end of first-line treatment and surgery. While exercise and education are cornerstones in both first-line treatment and prehabilitation, the aim of prehabilitation differs from that of first-line treatment. Prehabilitation aims to prepare patients for the upcoming TKR and optimise postoperative recovery, increase patients’ enablement and self-efficacy and reduce unnecessary healthcare use. However, evidence supporting prehabilitation to enhance postoperative recovery is limited and inconsistent and is largely based on low-quality, heterogeneous studies with insufficient description of interventions, control groups, progression models and adherence.^21^,^24^

A better understanding of how prehabilitation affects postoperative outcomes after TKR is needed. This trial aims to contribute to existing knowledge by investigating the combined effect of preoperative exercise therapy and patient education on postoperative outcomes, as these interventions are often studied separately in previous research. The trial also examines the impact of prehabilitation on postoperative patient-reported joint awareness and enablement, which has rarely been assessed. Additionally, objective measurement of physical activity using the activPAL accelerometer preoperatively and postoperatively provides novel insights into physical activity patterns in this population.

The aim of this trial is to investigate the effect of adding prehabilitation to standard care before TKR, compared with standard care alone, focusing on (1) patient-reported joint awareness 1 year after TKR, and (2) patient enablement and objectively measured knee function 6 weeks after TKR.

## Methods and analysis

### Trial design

The PRe-Operative exercise Therapy and Education before total Knee replacemenT (PROTEKT) trial is a multicentre parallel-group randomised controlled trial (RCT) planned to be conducted within two specialised orthopaedic outpatient rehabilitation units in the southeast healthcare region of Sweden, a region with both urban and rural patient catchment areas. This protocol is reported in accordance with the Standard Protocol Items: Recommendations for Interventional Trials (SPIRIT)^25^ and the Template for Intervention Description and Replication (TIDieR) checklist.^26^ Results will be reported in accordance with the Consolidated Standards of Reporting Trials (CONSORT) guidelines.^27^

### Patient recruitment and inclusion/exclusion criteria

**Figure 1:**
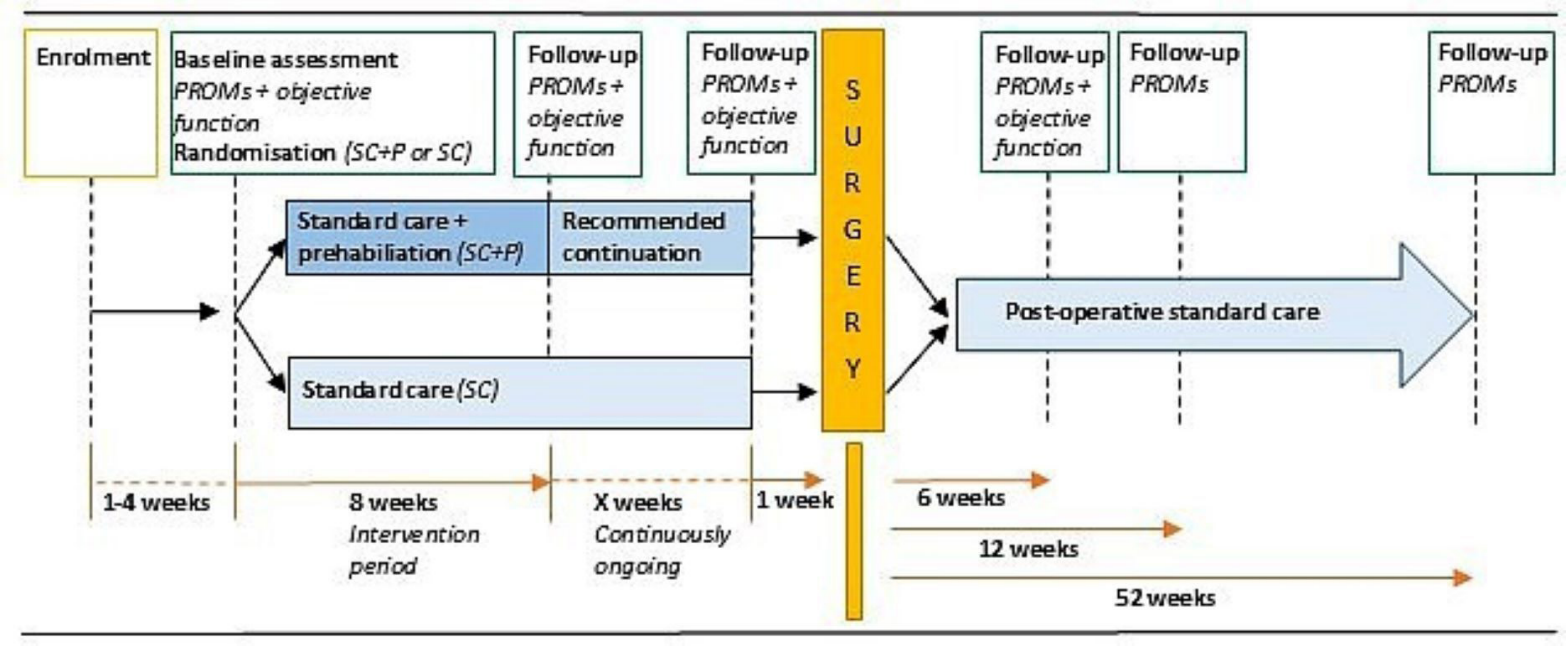
Trial timeline. PROMs, patient-reported outcome measures.

Participating centres contribute to patient recruitment using a stepwise approach, with one centre initiating recruitment first and the other following in the second stage. All patients referred to an orthopaedic surgeon at the participating hospitals for assessment of the need for TKR are screened for eligibility and receive written information about the trial by post from an administrator before the appointment. Eligible patients (table 1) receive additional oral information and are invited to participate in the trial by a nurse involved in the appointment. The nurse obtains signed informed consent (online supplemental file 1) and delivers it to the trial coordinator (lead author). Time for consideration is offered if needed. An appointment for baseline measures and randomisation is scheduled by the trial coordinator. A visual representation of the trial timeline is shown in figure 1.

**Table 1:** Inclusion and exclusion criteria

Inclusion criteria	Exclusion criteria
40–85 years at the time of surgery decision	Previous knee replacement in the other knee
Awaiting primary unilateral total knee replacement	Other reason than knee OA as the primary reason for surgery
Knee OA being the primary reason for surgery	Impaired cognitive function
Reside within 60 min travel time to the site of the intervention	Not fluent in written and spoken Swedish
	Chronic illness or disability, etc hindering full participation in the intervention

OA, osteoarthritis.

### Randomisation

Patients are randomised using opaque, concealed envelopes and stratified by age (≤67 years, >67 years) with a 1:1 allocation ratio, based on computer-generated random number sequences, into the intervention group, comprising prehabilitation in addition to standard care (SC+P), or the control group, consisting of standard care only (SC). The computer-generated random number envelope sequences were created by a person not otherwise involved in the trial. Allocation is performed by the trial coordinator immediately after the baseline assessment. The trial coordinator did not have access to the randomisation lists before allocation.

### Prehabilitation

Prehabilitation consists of 8 weeks of on-site, supervised and individualised exercise therapy together with patient education. It is delivered in a group format two times per week, at a fixed time in the afternoon (approximately 1 hour per session), by physiotherapists with experience in the rehabilitation of OA and TKR.

After the 8-week intervention, patients are advised to continue the exercise therapy either in supervised sessions or unsupervised at a location of their choice until the surgery date. Patients who choose to continue with unsupervised exercise therapy have check-ins scheduled by phone or video conference every other week with the physiotherapist in charge of the intervention to discuss exercise progression and adjustments.

Physiotherapists at the participating clinics are trained in the intervention’s content and structure two times before enrolling patients. They work with the trial coordinator to review these materials, and monthly meetings are held to monitor sessions, ensure treatment fidelity and address any issues.

### Exercise therapy

The exercise therapy content is based on recommendations for preoperative exercise therapy.^28^ The exercise protocol follows a standardised structure but is individually customised for each patient. It is divided into four sections with different exercise focus (warm-up, strengthening, balance and ROM exercises), and each exercise within each section has three difficulty levels (table 2). The content is designed and dosed to promote maximal strength development in multijoint strength exercises, muscle hypertrophy in single-joint strength exercises, improved balance and increased joint ROM.^29^,^31^

**Table 2:** Exercise therapy content

Exercise type		Exercise options	Difficulty level	Dosage, set × reps/time	Effort/intensity
Warm-up exercises	1	Stationary bike Seated bike Cross-trainer	N/A	10 min	RPE 11–12
Strengthening exercises	2	Bilateral leg press (full ROM) Bilateral leg press (partial ROM) Unilateral leg press (full ROM)	Main option Easier Harder	3×6 (primary) 3×8 (secondary) 3×10 (tertiary)	RPE 17–18
	3	Supine unilateral hip bridges Supine bilateral hip bridges Supine unilateral hip thrusts	Main option Easier Harder	3×6 (primary) 3×8 (secondary) 3×10 (tertiary)	RPE 17–18
	4	Bilateral seated leg extensions (machine) Unilateral seated leg extensions with ankle weights Unilateral seated leg extensions (machine)	Main option Easier Harder	3×12 (primary) 3×13 (secondary) 3×14 (tertiary)	RPE 17–18
	5	Bilateral seated leg curls (machine) Unilateral seated leg curls with resistance band Unilateral seated leg curls (machine)	Main option Easier Harder	3×12 (primary) 3×13 (secondary) 3×14 (tertiary)	RPE 17–18
	6	Standing bilateral calf raises Seated unilateral calf raises with weights Standing unilateral calf raises	Main option Easier Harder	3×12 (primary) 3×13 (secondary) 3×14 (tertiary)	RPE 17–18
Balance exercise	7	Static unilateral balance, level ground, no hand support Static unilateral balance, level ground with hand support Static unilateral balance, unstable surface, no support	Main option Easier Harder	3×30 s	Ability to maintain balance
Range of motion exercises	8	Supine active flexion slides Seated active flexion slides Supine active flexion slides with end-range assistance	Main option Easier Harder	5×5 s	Pain NRS ≤5 (0–10)
	9	Supine active knee extensions, heel elevated Supine active knee extensions Supine active knee extensions, heel elevated, extra weight	Main option Easier Harder	5×5 s	Pain NRS ≤5 (0–10)

N/A, not applicable; NRS, numerical rating scale; ROM, range of motion; RPE, rate of perceived exertion.

The Borg scale for rating of perceived exertion (RPE)^32^ is used to monitor exercise effort in strengthening exercises, with an RPE of 17–18 as the target. Pain response during knee ROM and strengthening exercises is monitored using a numerical rating scale (NRS) of 0–10.^33^,^36^ Pain intensity is allowed to increase by up to five points from the start of the exercise and should subsequently decrease by at least two points before initiating the next set or exercise. In balance exercises, the ability to maintain balance during a set is used to monitor effort.

The rest period between sets or exercises is not standardised. Patients are instructed to rest for as long as needed for fatigue and/or pain to settle sufficiently (as described above) before starting the next set or exercise. Based on this, rest periods are typically between 1 and 3 min.

#### Exercise progression

Exercises are progressed when patients can complete the prescribed dosage without an unacceptable pain response, as described in the previous section.^33^,^36^ In strengthening exercises, linear progression of exercise intensity by the smallest possible increment in each exercise is the primary progression method, but if linear progression is not suitable, progression is achieved by increasing volume through additional repetitions (3×6 → 3×8 → 3×10 or 3×12 → 3×13 → 3×14 → 3×15), adding exercises or increasing exercise difficulty. In ROM exercises, progression is mainly achieved by increasing the exercise difficulty level or by increasing end-range stretching amplitude (allowed pain intensity NRS ≥5). In balance exercises, when the patient can maintain balance with ease, exercises are progressed by increasing exercise difficulty (table 2).

#### Exercise individualisation

The exercises are individualised primarily by selecting the exercise difficulty level (easier, main or harder option) but can also be tailored by adjusting the total number of exercises in the protocol (minimum four exercises, maximum nine exercises, including warm-up) (table 2).

An individual assessment of previous exercise habits is conducted for each patient at baseline, before designing the initial exercise therapy protocol. Patients are asked about their level of physical activity, exercise and sedentary time over the previous month, and whether they currently perform any exercises targeting the knee or lower extremities. Patients who report regularly performing lower extremity strengthening exercises (at least once per week) can begin at a higher level (harder exercise options or more exercises) compared with patients who do not engage in regular lower extremity strengthening exercises.

#### Knee health monitoring and training load adjustments

Before each session, patients assess their overall knee health using the Single Assessment Numerical Evaluation (SANE) score and record it in the exercise protocol. The SANE score is a patient-reported rating scale in which respondents assess their knee health on a 0–100 scale, with 100 representing full health and 0 the worst possible health.^37^ Patients answer the question, ‘How do you rate your overall knee health today?’ The SANE score has been validated against commonly used PROMs (patient-reported outcome measures) and is suitable for assessing overall knee health in daily clinical practice for patients undergoing TKR.^38^

The SANE assessments are monitored throughout the entire intervention. Training loads are adjusted if patients report worsening knee health over three consecutive sessions. Load reduction is primarily achieved by decreasing intensity, but volume or exercise difficulty may be reduced if lowering intensity is not appropriate. Conversely, if patients report stable or improved knee health over time, training loads are progressed as planned.

#### Patient education

The patient education consists of individual conversations between the physiotherapist and the patient on various topics, selected based on consensus in previous literature.^28^ The topics are presented in box 1. Conversations take place during exercise sessions, either during the warm-up, between sets or at the end of the session, and may involve one-on-one discussions or multiple patients at the same time. Conversations typically begin with questions posed by the physiotherapist, for example, ‘What are your thoughts/expectations of…?’ or with questions from the patients. The conversation format is not standardised but is adapted according to the situation.

Box 1 **Education topics**

- Purpose of prehabilitation.
- The pain monitoring model.
- Pain and swelling with exercise and load.
- Postoperative course of events.
- Postoperative rehabilitation.
- Postoperative pain and swelling.
- Expectations of future function/ability.
- Expectations of future activities/activity level.
- Other.

The aim of the conversation is not to convince or instruct patients on what their expectations should be, but to create a nuanced and realistic understanding of what to expect during recovery after TKR and to individually tailor the content to each patient. All education topics are discussed with each patient on at least two occasions. Patients are encouraged to reflect on the conversations until the next session and to ask follow-up questions if needed. If the same ‘other topic’ arises in conversations with multiple patients, it is considered a main topic and will be included in discussions with all patients going forward.

### Standard care

Before surgery, standard care consists of self-care and a single, standardised 1.5-hour preoperative education session, delivered approximately 2–3 weeks before surgery. General oral and written information is provided regarding preparations for surgery (eg, preparing the home environment), events during the hospital stay and initial postoperative rehabilitation. No specific preoperative exercise advice is given. The standardised preoperative education session is delivered by healthcare staff (physiotherapist, occupational therapist, nurse) who are not involved in the present trial.

During the hospital stay (1–2 overnights) after surgery, all patients are instructed in and begin a standardised home-based postoperative rehabilitation protocol. Instruction is provided by physiotherapists at the orthopaedic clinic, with follow-up conducted by a primary care physiotherapist. All patients are scheduled for a 6-week post-surgery assessment by a physiotherapist at the orthopaedic clinic. Following this assessment, patients in both groups return to their primary care physiotherapist and continue rehabilitation based on their individual goals for as long as necessary. No further follow-up assessments are scheduled at the orthopaedic clinic.

### Data collection and blinding

A questionnaire covering patient demographics, lifestyle behaviours, expectations and PROMs is mailed to patients, completed and brought to the baseline assessment, where objective function measures are conducted. At the baseline assessment, a triaxial accelerometer (activPAL) is attached to the front of the thigh of the affected leg and worn 24 hours a day for 7 days. The accelerometer is then returned to the trial coordinator using a pre-paid reply envelope.

Follow-up assessments are conducted immediately after the 8-week intervention and at 6, 12 and 52 weeks post-surgery. Waiting times for surgery will vary between patients, and if more than 4 weeks elapse between the post-intervention follow-up and surgery, an additional follow-up assessment will be scheduled approximately 1 week before surgery.

PROMs are collected at every follow-up assessment. Measures of objective function are conducted at the 8-week follow-up, 1 week before surgery and 6 weeks after surgery. The activPAL accelerometer is worn 24 hours a day for 7 days immediately after the 8-week follow-up and at the 12-week and 52-week postoperative follow-up assessments.

Outcome data are collected locally at each participating centre. All PROMs in the trial are administered via paper surveys. Measures of objective function are assessed by a physiotherapist blinded to group allocation. Physiotherapists performing these assessments are trained on two occasions before participating in the trial, during which pilot measurements of objective outcome measures are conducted under the supervision of the trial coordinator. The assessors also participate in monthly meetings with the trial coordinator throughout the trial to address potential issues.

Patients are not blinded to group allocation but are not informed of the trial hypothesis until data collection is complete. An overview of the trial timeline and data collection is provided in figure 1 and table 3.

**Table 3:** Data collection/outcome measures

Data collection time points	Baseline	8-week follow-up	1-week preop follow-up (if necessary)	6-week postop follow-up	12-week postop follow-up	52-week postop follow-up
Demographics, lifestyle behaviours, expectations, etc	X					
PROMs						
FJS-12	X	X	X	X	X	**X**
Satisfaction						X
Mod-PEI-2	X	X	X	**X**	X	X
KOOS	X	X	X	X	X	X
IPAQ-sf	X	X	X	X	X	X
EQ5D-3L	X	X	X	X	X	X
Objective function						
Isometric quadriceps strength	X	X	X	**X**		
Active knee ROM	X	X	X	**X**		
30s CST	X	X	X	**X**		
activPAL accelerometer	X	X			X	X

Bold crosses are considered main outcome time points.

EQ5D-3L, EuroQol 5 Dimension 3 Levels; FJS-12, Forgotten Joint Score-12; IPAQ-sf, International Physical Activity Questionnaire-short form; KOOS, Knee injury and Osteoarthritis Outcome Score; Mod-PEI-2, modified Patient Enablement Instrument 2; postop, postoperation; preop, preoperation; ROM, range of motion; 30s CST, 30 second Chair to Stand-Test; w, week.

### Outcome measures

An overview of outcome measures collected at each time point is provided in table 3.

#### Primary outcome measures

##### Forgotten Joint Score-12

Patients assess their knee joint awareness using the Forgotten Joint Score-12 (FJS-12), which consists of 12 items rated on a 5-point Likert scale in different daily living situations. The results are summarised into a total score on a 0–100 scale, with 0 indicating worst (high joint awareness) and 100 indicating best (low joint awareness).^15^ Lower awareness of the operated joint is considered to reflect higher postoperative satisfaction. Psychometric properties of the FJS-12, including minimal clinically important difference (MCID), minimal important change (MIC), minimal detectable change and patient-acceptable symptom state thresholds, have been previously determined using patient satisfaction with surgery (5-point Likert scale: very satisfied, satisfied, neither satisfied nor dissatisfied, dissatisfied, very dissatisfied) as an anchor.^16^ The FJS-12 demonstrates good validity and reliability, with low floor and ceiling effects and is well suited for a population of Swedish patients undergoing TKR.^39 40^

##### Modified Patient Enablement Instrument-2

The modified Patient Enablement Instrument-2 (Mod-PEI-2) is based on the PEI-2, a 6-item PROM in which patients assess their ability to cope with life, understand and manage their disease, maintain health and help themselves. In the original version, patients respond based on information received at a prior doctor’s appointment, whereas in this modified version, patients evaluate their coping abilities over the previous 4 weeks, regardless of any intervention received. Items are rated on a 5-point Likert scale, ranging from ‘extremely well’ to ‘not at all’. Scores are summed to give a total ranging from 6 to 30, with higher scores indicating greater enablement.^41^ Both the Mod-PEI-2 and the Swedish version of the original instrument have demonstrated good validity, reliability, sensitivity and responsiveness, making them suitable for use in intervention studies.^42 43^

### Secondary outcome measures

#### Patient satisfaction 52 weeks after surgery

52 weeks after surgery, patients are asked to respond to the question, ‘How satisfied are you with the results of your surgery?’ using a single-item, 5-category Likert scale with the following response options: very satisfied, satisfied, neither satisfied nor dissatisfied, dissatisfied or very dissatisfied. This single-item measure of satisfaction has been tested in a large-scale arthroplasty registry and is internationally recognised as a valid and relevant measure of patient satisfaction.^44 45^

#### Patient-reported function and health-related quality of life

Patient-reported function and health-related quality of life are assessed using the Knee injury and Osteoarthritis Outcome Score (KOOS) and the EuroQol 5 Dimensions 3 Levels (EQ-5D-3L) questionnaires. KOOS has demonstrated adequate measurement properties across different knee injuries and age groups, including older adults with knee OA undergoing TKR.^46 47^ The EQ-5D-3L shows good reliability and validity but is less responsive than the 5L version in patients undergoing TKR.^48^

#### Physical activity and sedentary habits

Physical activity is assessed using the short version of the International Physical Activity Questionnaire (IPAQ-SF). While patient-reported outcomes have limitations in measuring physical activity and sedentary behaviour—patients tend to under-report low-intensity activities and sedentary behaviour and over-report higher-intensity activities—the IPAQ-SF is considered an acceptable tool for patients with knee OA, where the collection of patient-reported outcomes is feasible.^49 50^

Objective physical activity is measured using the activPAL accelerometer. The device is attached with waterproof tape to the front of the affected thigh and worn 24 hours a day for 7 consecutive days. Using the activPAL accelerometer to collect objective measures of physical activity is a feasible method that provides acceptable accuracy for estimating activity levels in free-living individuals.^51 52^

#### Objective function

Isometric quadriceps strength is measured with the patient seated in 90° knee flexion using a fixed dynamometer (Meloq Easyforce, product no. 2009002), without back support, and with hands resting on the plinth. Following instructions, patients familiarise themselves with the test by performing two submaximal repetitions, followed by two maximal repetitions of three seconds each on both legs, starting with the non-affected leg. The highest value achieved (in Newtons) for each leg is recorded as the result. Isometric testing of quadriceps strength is suitable for patients undergoing TKR and may be associated with lower pain during testing compared with isotonic testing.^53^

Active knee range of motion is measured with the patient supine using a long-arm goniometer. The maximal active, unassisted extension and flexion angles are recorded for each leg, starting with the non-affected leg. This method of measuring active knee ROM is reliable and provides acceptable accuracy, although it tends to slightly underestimate knee flexion angles.^54 55^

Functional performance is assessed using the 30 seconds Chair to Stand-Test (30s CST), in which the patient completes as many sit-to-stand repetitions as possible in 30 seconds from a standard-height chair (45 cm) with arms crossed over the chest. The 30s CST is recommended by the Osteoarthritis Research Society International for patients with knee OA and demonstrates good reliability and validity.^56^,^58^

### Other measures

#### Adherence

Adherence to the intervention is recorded using an exercise journal maintained by the physiotherapist overseeing the intervention. Patients’ attendance and participation in each session are documented, along with the education topics discussed with each patient during every session. The journal tracks the number of exercises performed, variation of exercises, dosage and load used and the SANE score for each patient at every session. Educational topics discussed are recorded individually for all patients, although the detailed content of these discussions is not documented. Patients without a scheduled surgery date after the 8-week intervention who choose to continue exercise therapy, either supervised or unsupervised, maintain an identical exercise journal and record their own sessions. To support continued adherence, check-ins by phone or video conference with the physiotherapist in charge are scheduled every other week, as previously described.

For patients assigned to standard care, any participation in ongoing exercise interventions outside the trial is recorded using patient-reported forms at baseline and at the 8-week and 1-week pre-surgery follow-ups. This includes the type of exercise performed, its duration and weekly frequency. Participation in the standardised preoperative education session is also documented.

#### Description of surgical procedures and postoperative physiotherapy

Information about the surgical procedure, including type of replacement, use of patella button/resurfacing and any soft tissue procedures beyond standard practice, is collected from the surgery report. Anthropometric data, such as weight, height, body mass index and American Society of Anesthesiologists (ASA) physical status classification, are obtained from the preoperative anaesthesiologist assessment.

Physiotherapy care is not standardised after the initial 6-week postoperative programme; therefore, a brief description of postoperative physiotherapy management is collected at the 6-week, 12-week and 52-week postoperative follow-up assessments.

#### Adverse events

Adverse events, including injuries sustained during intervention participation, re-surgeries (with reasons), postoperative infections and deaths, are recorded for all patients.

### Data management and patient confidentiality

All digital trial data are stored on a secure server managed by Region Östergötland. All physical trial documentation (envelopes, surveys, exercise protocols, etc) is stored in a fireproof locker accessible only to the research group. Patients are assigned a unique three-digit ID, and the key code is accessible only to the trial coordinator. Only coded data are shared among the authors in the research group.

### Sample size

To detect a difference in the MIC of 17.7 points (effect size 0.61) and the MCID of 13.7 points between the SC+P and SC groups in the FJS-12^16^ from baseline to the 52-week follow-up, and assuming a 0.6 proportion of total scores >21 points for the Mod-PEI-2 in the SC+P group versus 0.3 in the SC group at the 6-week follow-up, a total of 110 patients is targeted for inclusion across the two groups (80% power, 5% α-level, with an estimated 20% dropout included).

### Analysis plan

All analyses will be performed using IBM SPSS Statistics software, V.29, applying intention-to-treat and per-protocol approaches with an α-level of 0.05. Demographic data will be presented as mean or median with SD or IQR for quantitative variables, and as frequencies and percentages for categorical variables.

Depending on measurement scale and distribution, differences between groups at baseline and for primary and secondary outcomes at each follow-up assessment will be analysed using independent samples t-tests or non-parametric equivalent tests. Longitudinal changes within groups from baseline to follow-up time points will be analysed using linear mixed models or generalised linear models, with baseline measurements included as covariates. Multiple linear regression and logistic regression will be used to analyse predictor variables for the primary outcomes (FJS-12 and Mod-PEI-2). Furthermore, analyses of primary and secondary outcomes will be adjusted, if necessary, for differences in intervention delivery and adherence, duration of participation, surgical procedures and postoperative physiotherapy care.

Multiple analyses will be performed using α-level adjustment methods, such as the Bonferroni method. In the intention-to-treat analysis, a sensitivity analysis will be performed to quantify the magnitude of missing data from patients lost to follow-up. Appropriate statistical methods will be used to account for missing data in the analysis, depending on the characteristics of the missing data. If there is extensive missing data, a second table of baseline characteristics will be presented only for patients included in the final analysis. The proportion of missing outcomes per group, characteristics of patients with missing data and possible reasons for missing outcome data will be presented.

### Patient and public involvement

Patients with knee OA were involved in the early planning stage of this trial by providing their perspectives on the time frame from referral in primary care to consultation with the orthopaedic surgeon and subsequent surgery. This input was used to inform the planning of the intervention. After completion, the RCT will be followed by a qualitative interview study with participants from the intervention group to explore the feasibility of implementing the intervention in clinical practice.

## Ethics and dissemination

The trial has received ethical approval from the Swedish Ethical Review Authority (reg. no. 2023-05120-01) and is pre-registered at ClinicalTrials.gov (trial no. NCT06290336). The trial complies with the Declaration of Helsinki. It is funded by the Medical Research Council of Southeast Sweden, which has not been and will not be involved in planning, conducting, analysing or presenting the results. Signed informed consent is obtained from all patients before participation in the trial (online supplemental file 1).

Dissemination of aggregated trial results to relevant professional and patient interest groups will be conducted through conference presentations, meetings and submission for publication in a peer-reviewed journal. The findings may influence future clinical guidelines and care pathways for patients undergoing TKR.

### Trial status, anticipated time frame and changes to the protocol

All patients booked for an initial assessment by an orthopaedic surgeon receive letters of invitation together with written trial information 1–2 months prior to the scheduled visit. The letters and trial information began to be sent out in December 2023, and the first eligible patient was recruited on 26 February 2024. Recruitment is expected to continue throughout 2026, and subsequent data collection is anticipated to be completed by the end of 2027.

Patient satisfaction with the results 52 weeks after surgery was changed from a primary to a secondary outcome after the initiation of the trial, as satisfaction is closely linked to and captured by the current primary outcome measure, FJS-12.^15 16^ Furthermore, after recruitment of the first 22 patients, measurement of objective physical activity using the activPAL accelerometer was added to the secondary outcomes to supplement the existing physical activity PROMs.

## Discussion

### Strengths and limitations

Strengths of this trial include the following: (1) concealed, stratified randomisation to reduce the risk of allocation bias; (2) supervised exercise therapy, allowing easy monitoring of exercise technique, dosage and progression; (3) blinded assessors of objective outcomes to reduce the risk of detection bias; (4) multicentre design to increase external validity; (5) individualisation of total exercise number and variations, enabling tailored exercises and promoting person-centred care; (6) intervention delivered in a group format, offering peer support; (7) combination of exercise therapy and patient education according to recommendations, improving functional performance and creating realistic expectations among patients awaiting TKR; and (8) opportunity to ask individual questions and engage in discussion with physiotherapists experienced in preoperative and postoperative rehabilitation of TKR.

Limitations of this trial include the following: (1) waiting time to surgery will differ between patients, resulting in variable total exposure time and challenges for statistical analysis; (2) the combined intervention design does not allow differentiation between the effects of exercise therapy and patient education; (3) on-site intervention delivery at a set time in the afternoon may exclude patients with long travel distances and make participation difficult for working-age patients or those with other commitments; (4) individualisation of exercise therapy and patient education may limit comparability with other studies and generalisability of results, although individualisation is performed within predetermined boundaries; (5) patients’ expectations may increase as a result of the intervention, potentially raising the risk of dissatisfaction if expectations are not fulfilled; (6) satisfaction is a multifactorial concept and challenging to assess comprehensively^59 60^; and (7) the trial coordinator (primary investigator) and statistician are not blinded to group allocation.

### Potential impact on future clinical guidelines

There is a gap in the chain of care for patients with OA when first-line treatment in primary care is no longer sufficient and TKR is the next stage. Patients often discontinue exercise therapy, leading to increased inactivity,^18^ while waiting times for surgery in Sweden can extend for many months, sometimes exceeding a year. During this waiting period, patients are often left to manage on their own, increasing the risk of inactivity and deterioration of knee function. This period presents an opportunity for improved management of these patients and may increase the likelihood of better outcomes after TKR.

By providing a well-designed RCT with sufficient intervention length and thorough description of exposure for both the intervention and control groups, this trial addresses common flaws in previous prehabilitation studies, such as lack of intervention detail, progression models and adherence monitoring. Furthermore, it provides novel insights by investigating the effect of preoperative exercise combined with patient education on postoperative outcomes, rather than examining only one component. The trial also examines the impact of prehabilitation on postoperative patient joint awareness and satisfaction and provides important information on physical activity patterns in this population through objective measurements using the activPAL accelerometer, both preoperatively and postoperatively. Hence, this trial will contribute to current knowledge regarding the efficacy of prehabilitation before TKR to improve postoperative outcome, specifically focusing on patient-reported joint awareness, satisfaction, enablement and knee function.

## Supplementary material

10.1136/bmjopen-2025-113185 online supplemental file 1
