# Supplementary material for: Effect of prehabilitation before total knee replacement on postoperative patient-reported joint awareness, enablement and knee function: protocol for the PROTEKT randomised controlled trial
Source: BMJ Open. 2026 Mar 3;16(3):e113185. doi: 10.1136/bmjopen-2025-113185 (PMC12958917; doi:10.1136/bmjopen-2025-113185)
Supplement: online supplemental file 1 [file bmjopen-16-3-s001.docx]

# Prehabilitation before total knee replacement

**Information for Research Participants**

We would like to ask if you would like to participate in a research project. In this document, you will find information about the project and what participation involves.

**What is this project, and why do you want me to participate?**

Osteoarthritis is a widespread disease, and its prevalence is increasing as the population grows. The condition is primarily treated with non-surgical interventions, but for some patients, these measures are not sufficient, and total knee replacement becomes necessary. Undergoing knee replacement surgery and the following rehabilitation is a long and demanding process for the patient. It takes time to achieve satisfactory knee function after the operation—sometimes up to a year—and the results largely depend on the patient’s own efforts during rehabilitation.
The purpose of this research project is to examine how training and patient education before surgery affect the patient’s perception of the knee and its function afterwards. By participating in this project, you contribute to improving healthcare’s ability to support patients in an increasingly common health issue.

The research principal for the project is Region Östergötland. The research principal is the organization responsible for the project. The application has been approved by the Swedish Ethical Review Authority, reference number 2023-05120-01.

**How does the project work?**

Patients undergoing total knee replacement for the first time and who meet predetermined criteria will, after the surgical decision, be randomly assigned to either a study group or a control group.

Participants in the study group will attend the physiotherapy department at the Orthopedic Clinic’s rehabilitation unit in Norrköping and take part in training and patient education led by a physiotherapist twice a week, starting as soon as possible after the surgery decision and continuing until the surgery. The training includes strength and mobility exercises and, together with the education, aims to prepare the patient for the operation and postoperative rehabilitation. The training and education will be conducted in group format and follow a shared structure but will be individualized according to the participants' abilities. Each session will last approximately one hour. Participants will undergo physical assessments and complete questionnaires after the surgical decision, 8 weeks after the first measurement, 1–2 weeks before surgery, and 6 weeks after surgery. Questionnaires will also be completed 3 months and 1 year after the operation.

Study group participants may also be invited to participate in an interview after the final follow-up one year post-surgery. The interview will focus on participants’ experiences of the intervention and how it may have influenced their rehabilitation. The interview is expected to take about 30–60 minutes.

Participants in the control group will continue according to the standard care program. This includes being invited to an information meeting about 2–3 weeks before surgery and undergoing routine rehabilitation and follow-up afterwards. They will also undergo physical assessments and complete questionnaires after the surgical decision, 8 weeks after the first measurement, 1–2 weeks before surgery, and 6 weeks after surgery, as well as questionnaires 3 months and 1 year after the operation.

All participants will wear an activity monitor on the thigh for one week at the start of the study, for one week during the 8‑week follow-up measurement, and again at 3 months and 1 year after surgery. The device is small, does not interfere with activity, and is not visible under normal clothing. It is worn day and night and does not need to be removed for showering or hygiene.

**Possible consequences and risks of participating**

Physical training for knee osteoarthritis may temporarily increase symptoms such as pain, discomfort, swelling in the knee, and muscle soreness in the surrounding musculature. These effects are temporary, and training has not been shown to have negative effects on the osteoarthritis itself, even with high-load exercises or in advanced stages of the condition.

If your symptoms increase, you may continue participating in the training, but the program will be adjusted to your comfort level.

All patients, regardless of group, will undergo measurements of thigh muscle strength, knee joint mobility, and sit-to-stand ability. These assessments may also cause temporary increases in joint pain, discomfort, swelling, and muscle soreness. The risk of injury during these assessments is very low, and the procedures are adapted to minimize risk.

**What happens to my data?**

The project will collect and register information about you. This includes measurement results of your strength and mobility, your questionnaire responses, and general details about your medical background such as height, weight, and concurrent medical conditions.

All collected data will be stored on Region Östergötland’s server. Your responses and results will be handled securely and coded/anonymized so unauthorized individuals cannot access them. Once collected, the data will be compiled and published in national and international scientific journals. The report will not include individual results—only group-level data. All project staff are bound by confidentiality, meaning they may not disclose information about individual participants.

Region Östergötland is responsible for your personal data. According to the EU General Data Protection Regulation (GDPR), you have the right to access the data about you, and to request corrections if necessary. You may also request deletion of your data or restriction of processing. However, the right to deletion or restriction does not apply if the data is necessary for the research.
If you wish to access your data, contact the project management (see below). The Data Protection Officer can be reached at dataskyddsombud@regionostergotland.se. If you are dissatisfied with how your data is handled, you may file a complaint with the Swedish Authority for Privacy Protection (IMY). The study is approved by the Swedish Ethical Review Authority. If you have questions about the study, you are welcome to contact the project management.

**How will I receive information about the results?**

Results of the project will be published in relevant scientific journals. If you wish to receive your personal results from the study, you may contact the project management.

**Insurance and fees**

Participants are covered by the standard patient injury insurance applicable to all healthcare treatment. Normal patient fees (200 SEK) and the high-cost protection (1400 SEK) apply for your scheduled visits.

**Voluntary participation**

Your participation is voluntary, and you may withdraw at any time. You do not need to state a reason, and your decision will not affect your future care or treatment.

If you wish to withdraw, please contact the project leader (see below).

**Project Contacts**

Joanna Kvist, Professor of Physiotherapy
Division of Physiotherapy, Department of Health, Medicine and Caring Sciences (HMV), Linköping University
Principal Investigator
Joanna.kvist@liu.se

Marcus Ljung, Licensed Physiotherapist
PhD student at the Division of Physiotherapy, HMV, Linköping University
Project manager and contact person at the Orthopedic Clinic, Vrinnevi Hospital, Norrköping
Phone: 010-1043861
Marcus.Ljung@regionostergotland.se

Kristin Gustafsson, Specialist Physiotherapist in Orthopedics
PhD at the Division of Physiotherapy, HMV, Linköping University

**Consent to Participate in the Project**

I have received oral and/or written information about the study and have had the opportunity to ask questions. I may keep the written information. It is possible to consent only to the first part or to both parts of the study.

- I consent to participate in the project «Prehabilitation before total knee replacement»

| Place and date | Signature |
| --- | --- |
|  |  |
|  | Printed name |
|  |  |

- I consent to participate in an interview after the final follow-up measurement of the project

| Place and date | Signature |
| --- | --- |
|  |  |
|  | Printed name |
|  |  |
