# Supplementary material for: Effect of prehabilitation before total knee replacement on postoperative patient-reported joint awareness, enablement and knee function: protocol for the PROTEKT randomised controlled trial
Source: BMJ Open. 2026 Mar 3;16(3):e113185. doi: 10.1136/bmjopen-2025-113185 (PMC12958917; doi:10.1136/bmjopen-2025-113185)
Supplement: Supplementary file 2 [file bmjopen-2025-113185.reviewer_comments.pdf]

PEER REVIEW HISTORY

BMJ Open publishes all reviews undertaken for accepted manuscripts. Reviewers are asked to complete a checklist review form and are provided with free text boxes to elaborate on their assessment. These free text comments are reproduced below.

ARTICLE DETAILS

Title (Provisional)

Effect of prehabilitation before total knee replacement on post-operative patient-reported joint awareness, enablement and knee function: protocol for the PROTEKT randomised controlled trial

Authors

Ljung, Marcus; Gustafsson, Kristin; Kvist, Joanna

VERSION 1 - REVIEW

**Reviewer**	**1**
**Name**	**Cheok, Tim**
**Affiliation**	**Flinders University**
**Date**	**29-Nov-2025**
**COI**	**None**

1. Control group is not a constant. Authors should standardise "standard care"

2. I have some concerns on concealed envelope technique. This leads to allocation bias

---

**Reviewer**	**2**
**Name**	**Sezgin , Erdem A**
**Affiliation**	**Gazi University Faculty of Medicine, Orthopedics and Traumatology**
**Date**	**05-Jan-2026**
**COI**	**None**

The manuscript describes a robust protocol for an RCT addressing a relevant topic. The study's specific focus on resistance training combined with a structured "Patient Enablement" education model is a significant strength, aiming to overcome the limitations of previous under-dosed studies. The protocol is highly actionable and, if successful, offers a manualized prescription that is clinically distinct from generic prehabilitation.

Being a very well written protocol paper, I believe it can be accepted without revisions. However, I would like to note some structural risks that may ultimately have a negative effect on the study's eventual success. The most significant threat to internal validity is the temporal disconnect between the 8-week intervention and the surgery date. In healthcare systems with variable waiting times, physiological adaptations from the intervention may wash out before surgery if the wait extends months beyond the intervention. While the protocol recommends continued unsupervised exercise, adherence in this demographic is historically variable. Consequently, the "gap duration" becomes a critical covariate that must be strictly managed or analyzed to prevent the intervention group from becoming physiologically identical to the control group at the time of incision.

The sample size calculation appears vulnerable to underestimation given several design features that may dilute the treatment effect and obscure the intervention's value. Specifically, the "standard care" control group receives a standardized education session; if this includes exercise advice, they effectively become an active comparator, shrinking the potential delta between groups. Furthermore, the strict requirement for 8 weeks of on-site supervision within a 60-minute travel radius introduces significant selection bias, likely favoring wealthier, retired, or highly motivated participants. This "healthy user effect" risks compressing the between-group difference, as control patients drawn from this motivated pool may self-manage effectively while intervention patients might have exercised regardless. These diluting factors suggest the true effect size may be smaller than the optimistic estimate relying on MIC of 17.7 points or MCID of 13.7 points on the FJS-12 used for power calculations. This is critically important because the intervention is resource-intensive (16 supervised sessions); a merely "statistically significant" difference could be perceived as clinical noise and fail to justify the costs to payers, highlighting the need for a sample size robust enough to confirm a large, economically defensible effect. Also, if the true effect size is smaller, the current sample size risks a Type II error, potentially leading to a "no effect" conclusion despite clinical benefit.

Addressing these pragmatic and statistical risks now, if possible, perhaps by refining the "gap" analysis or reconsidering the optimistic effect size assumptions, can significantly strengthen the study's contribution to the literature.

VERSION 1 - AUTHOR RESPONSE

Reviewer: 1

Dr. Tim Cheok, Flinders University
Comments to the Authors:
1. Control group is not a constant. Authors should standardise "standard care".

Thank you for highlighting this concern. What is described as standard care in this trial is the same as the current care pathway for patients undergoing total knee replacement at the participating centres. This is for the most part consisting with care pathways for orthopaedic clinics in Sweden in

general. However, not all patients receive exactly the same 'standard care', individual differences occur due to different patient needs.

We describe the content of standard care under section '2.5 Standard care' on page 10 in the manuscript.

No further action taken.

2. I have some concerns on concealed envelope technique. This leads to allocation bias.

Thank you for your concern on this topic. In the allocation process, stratified randomisation (≤ 67 years of age and > 67 years of age) through sealed, opaque envelopes with computer-generated number sequences was used in order to reduce risk of allocation bias. To clarify the integrity and concealment of the allocation process, the following sentence was added in the manuscript in the randomisation section on rows 123-126:

"The computer-generated random number envelope sequences were created by a person not otherwise involved in the trial…The trial coordinator did not have access to the randomisation lists before allocation"

Reviewer: 2

Dr. Erdem A Sezgin , Aksaray University
Comments to the Authors:
The manuscript describes a robust protocol for an RCT addressing a relevant topic. The study's specific focus on resistance training combined with a structured "Patient Enablement" education model is a significant strength, aiming to overcome the limitations of previous under-dosed studies. The protocol is highly actionable and, if successful, offers a manualized prescription that is clinically distinct from generic prehabilitation.

Thank you for taking your time to review our manuscript for the ongoing PROTEKT-trial. We appreciate your comments, and your thoughtful concerns raised. This provides us valuable feedback on how to strengthen our manuscript, and relevant points for the future statistical inference and discussion. You have provided us with is valuable insights for the work ahead of us!

Being a very well written protocol paper, I believe it can be accepted without revisions. However, I would like to note some structural risks that may ultimately have a negative effect on the study's eventual success. The most significant threat to internal validity is the temporal disconnect between the 8-week intervention and the surgery date. In healthcare systems with variable waiting times, physiological adaptations from the intervention may wash out before surgery if the wait extends months beyond the intervention. While the protocol recommends continued unsupervised exercise, adherence in this demographic is historically variable. Consequently, the "gap duration" becomes a critical covariate that must be strictly managed or analyzed to prevent the intervention group from becoming physiologically identical to the control group at the time of incision.

Thank you for highlighting these concerns to us. We agree that we need to account for these in the upcoming analysis and following discussion. The "gap duration" described in your comment is a valid concern and has a potential to affect outcomes. This is the reason itself to why we see the need for an additional data collection time point for patients where more than four weeks passes after the 8-

week evaluation until the surgery. We will document waiting times for all participants, and take this into account in the analysis as described in the section ‘Analysis plan’ on row 396-398:

”Furthermore, analyses of primary and secondary outcomes will be adjusted, if necessary, for differences in intervention delivery and adherence, duration of participation, surgical procedures and post-operative physiotherapy care”.

This is also highlighted in the discussion section on rows 440-441:

“Waiting time to surgery will differ between patients, resulting in variable total exposure time and challenges for statistical analysis”

In the matter of adherence for patients continuing with unsupervised exercise after the 8 week intervention, adherence is measured as described in the ‘Adherence’ section on rows 350-351 (the ending of the sentence “…as described previously” was added for further clarification):

”To support continued adherence, check-ins by phone or video conference with the physiotherapist in charge are scheduled every other week as described previously”.

The sample size calculation appears vulnerable to underestimation given several design features that may dilute the treatment effect and obscure the intervention's value. Specifically, the "standard care" control group receives a standardized education session; if this includes exercise advice, they effectively become an active comparator, shrinking the potential delta between groups. Furthermore, the strict requirement for 8 weeks of on-site supervision within a 60-minute travel radius introduces significant selection bias, likely favoring wealthier, retired, or highly motivated participants. This "healthy user effect" risks compressing the between-group difference, as control patients drawn from this motivated pool may self-manage effectively while intervention patients might have exercised regardless. These diluting factors suggest the true effect size may be smaller than the optimistic estimate relying on MIC of 17.7 points or MCID of 13.7 points on the FJS-12 used for power calculations. This is critically important because the intervention is resource-intensive (16 supervised sessions); a merely "statistically significant" difference could be perceived as clinical noise and fail to justify the costs to payers, highlighting the need for a sample size robust enough to confirm a large, economically defensible effect. Also, if the true effect size is smaller, the current sample size risks a Type II error, potentially leading to a "no effect" conclusion despite clinical benefit. Addressing these pragmatic and statistical risks now, if possible, perhaps by refining the "gap" analysis or reconsidering the optimistic effect size assumptions, can significantly strengthen the study's contribution to the literature.

This is relevant criticism and wise thoughts. Firstly, patients do not receive specific exercise advise in the pre-operative information given in standard care. We have added a brief clarification on this topic on row 222-223:

“No specific pre-operative exercise advice is given”

Furthermore, the standardised pre-operative information given in standard care (to all patients) is delivered 2-3 weeks out from surgery, as stated in the manuscript (lines 219-220), making it unlikely to significantly affect results even if specific exercise advise were delivered.

Secondly, we are aware of that the requirement of 8 weeks of on-site intervention might affect the population studied in this trial. There is, as you point out, a risk that wealthier, resourceful patients might be more inclined to participate in this trial, and that e.g. patients of working age might be less

likely to participate. However, the intervention is set at a time in the late afternoon to promote participation for patients of working age, and in Sweden there are possibilities to apply for preventive sick leave to cover for loss of income. There are also possibilities to use subsidised, tax-funded, transportation back and forth to health care visits, promoting participation for less resourceful patients. All potential participants are informed about these possibilities before entering the trial. These factors might affect population studied in this trial, but it will not affect potential outcome differences between groups due to the inclusion criterions and stratified randomisation.

Thirdly, we agree that the sample size is calculated to significantly detect a rather large effect size, leaving the risk that smaller treatment effects might not achieve statistical significance and perceived as clinical noise, as you point out. However, this is based on previous research as to what is considered minimal relevant change in and difference between cohorts in the primary outcome Forgotten Joint Score-12 after total knee replacement. We argue that obtained differences less than these values (statistically significant or not) likely isn’t as clinically meaningful and is the reason to why we did not calculate sample size based on lower numbers. Furthermore, sample size calculation based on smaller effect sizes would have resulted in a total sample too large to manage within the trial logistics and realistic recruitment time frame.

VERSION 2 - REVIEW

**Reviewer**	**1**
**Name**	**Cheok, Tim**
**Affiliation**	**Flinders University**
**Date**	**04-Feb-2026**
**COI**	

No further concerns

**Reviewer**	**2**
**Name**	**Sezgin , Erdem A**
**Affiliation**	**Gazi University Faculty of Medicine, Orthopedics and Traumatology**
**Date**	**18-Feb-2026**
**COI**	

I have reviewed the revised PROTEKT protocol and the authors' responses. The authors have successfully addressed the administrative and clarity-related concerns, particularly regarding the alignment of the primary outcome (FJS-12) and randomisation concealment.

While my concerns regarding the "waiting list gap" and the optimistic power calculation remain, I acknowledge the authors' logistical constraints and accept their proposed mitigation strategies, including the statistical adjustment for wait times. This protocol

describes a well-structured investigation into prehabilitation concept that addresses a critical gap in the literature. Although the risk of a Type II error (due to detraining or high standard care quality) persists, it does not invalidate the study design at this stage.

Therefore, I believe the protocol is methodologically sound for publication. I look forward to the eventual results.
